# Schizophrenia-Associated hERG channel Kv11.1-3.1 Exhibits a Unique Trafficking Deficit that is Rescued Through Proteasome Inhibition for High Throughput Screening

**DOI:** 10.1038/srep19976

**Published:** 2016-02-16

**Authors:** Nicholas E. Calcaterra, Daniel J. Hoeppner, Huijun Wei, Andrew E. Jaffe, Brady J. Maher, James C. Barrow

**Affiliations:** 1 Departments of Pharmacology and Molecular Sciences, Baltimore, MD 21205; 2 Lieber Institute for Brain Development, Baltimore, MD 21205; 3 Johns Hopkins University Bloomberg School of Public Health, Baltimore, MD 21205; 4 Departments of Psychiatry and Behavioral Sciences, Baltimore, MD 21205; 5 Departments of Neuroscience, Johns Hopkins University School of Medicine, Baltimore, MD 21205

## Abstract

The primate-specific brain voltage-gated potassium channel isoform Kv11.1-3.1 has been identified as a novel therapeutic target for the treatment of schizophrenia. While this *ether-a-go-go* related K^ + ^channel has shown clinical relevance, drug discovery efforts have been hampered due to low and inconsistent activity in cell-based assays. This poor activity is hypothesized to result from poor trafficking via the lack of an intact channel-stabilizing Per-Ant-Sim (PAS) domain. Here we characterize Kv11.1-3.1 cellular localization and show decreased channel expression and cell surface trafficking relative to the PAS-domain containing major isoform, Kv11.1-1A. Using small molecule inhibition of proteasome degradation, cellular expression and plasma membrane trafficking are rescued. These findings implicate the importance of the unfolded-protein response and endoplasmic reticulum associated degradation pathways in the expression and regulation of this schizophrenia risk factor. Utilizing this identified phenomenon, an electrophysiological and high throughput *in-vitro* fluorescent assay platform has been developed for drug discovery in order to explore a potentially new class of cognitive therapeutics.

A principal goal of applying genetic association studies to exploring schizophrenia pathology has been to refocus drug discovery efforts in identifying novel therapeutic targets. Genetic loci significantly associated with schizophrenia can offer clues to the underlying etiology of this debilitating disorder that are largely unaffected by epiphenomena associated with illness. While these studies have resulted in a plethora of new insights into the genetic and epigenetic landscape associated with schizophrenia and psychiatric disorders, few proposed targets have resulted in biological and clinical validation^1^,^2^,^3^. One promising validated target initially found through genetic association studies is *KCNH2*, which codes for the voltage-gated potassium channel Kv11.1^4^.

In previous studies, single nucleotide polymorphisms (SNPs) in the second intron of the *KCNH2* gene have been associated with an increased risk of schizophrenia^5^. Additionally, meta-analysis of independent clinical association data sets have shown an association of these SNPs, with schizophrenia-relevant phenotypes, such as: lower IQ score, slower cognitive processing speed, decreased hippocampus gray matter volume, altered memory task fMRI signals, and positive patient response to olanzapine^6^. Furthermore, these risk-associated alleles associate with increased expression of a novel KCNH2 transcript, KCNH2-3.1, identified via mRNA characterization of post-mortem human brain tissues, suggesting a possible biological mechanism for the clinical associations^5^. Stemming from an alternative transcription start site, the KCNH2 3.1 transcript is missing the first two exons, resulting in an N-terminally truncated protein product, Kv11.1-3.1. By replacing the 102 amino acids coded by these two exons with 6 unique residues, Kv11.1-3.1 lacks most of the Per-Ant-Sim (PAS) domain present in the major isoform (Kv11.1-1A)^5^,^6^,^7^. It has been previously suggested that this alternate structure may be poorly folded, thereby decreasing channel stability (Fig. 1a)^8^. Furthermore, an intact PAS domain is essential for the slow deactivation rate typical of Kv11.1 channels, causing Kv11.1-3.1 to exhibit rapid deactivation along with altered channel gating kinetics. As a consequence, cells expressing Kv11.1-3.1 show reduced current accumulation during periods of constant depolarization and thus extended trains of action potentials. Therefore, increased expression of Kv11.1-3.1 channels may contribute to uncoordinated neuronal firing patterns in schizophrenia patients^9^.

The discovery of a small molecule that could selectively modulate Kv11.1-3.1 activity presents many challenges. The most apparent issue is channel homology to Kv11.1-1A (hERG), the primary channel involved in cardiac I_Kr_ currents and frequent anti-target causing arrhythmia, Long QT Syndrome, and torsades de pointes^10^. hERG channels have also been implicated in processes such as midbrain dopaminergic bursting and epilepsy, which may present unique challenges as Kv11.1-3.1 and Kv11.1-1A channels are expressed at similar levels in the brain and have been shown to form heterotetramers in cell models^5^,^8^,^11^,^12^. To develop novel modulators of this channel for psychiatric indications, great care must be taken to identify molecules with selectivity over the Kv11.1-1A form of the channel to avoid cardiac and neural toxicities. Robust screening methods with consistently large signals are required to rapidly discover promising tools; however, Kv11.1-3.1 presents significant obstacles to efficient screening^13^,^14^. While recording from cells overexpressing Kv11.1-3.1 is possible, previous studies have shown trafficking defects and low current densities comparable to Long QT syndrome (LQT) Kv11.1-1A mutants, which makes high throughput screening impractical^9^. Similar to Kv11.1-3.1, many of these LQT mutants contain mutations and deletions in the PAS domain, also resulting in rapid deactivation kinetics in HEK 293 cell models^10^,^15^,^16^,^17^. These kinetic effects are likely due to the loss of PAS domain interactions with the amphipathic helix containing N-cap and C-terminal nucleotide binding domain (cNBD)^18^,^19^,^20^. While it has been demonstrated that complete PAS domains are not necessary for trafficking or formation of channel heterotetramers, including Kv11.1-1A and Kv11.1-3.1 heterotetramers, a partial or mutated PAS domain leads to an instable PAS structure and subsequent trafficking deficiency^8^. Molecular rescue of these defects would facilitate efforts to create such a screening platform, and many examples of possible rescue techniques have been reported and characterized^15^,^21^,^22^,^23^,^24^,^25^,^26^. Nevertheless, no successful method of rescuing Kv11.1-3.1 trafficking has been reported to date.

Efforts were taken to describe the extent of expression or trafficking defects while simultaneously applying methods to attempt rescue, compatible with HTS assay design. Here it is demonstrated that Kv11.1-3.1 shows poor steady-state expression, low channel activity, and trafficking deficits. Pharmacological agents may be used to rescue these phenotypes via blockade of proteasomal activity. Utilizing this technique, an improved channel assay signal amenable to HTS is possible.

## Materials and Methods

### Molecular Biology

C-Terminally hemagglutinin (HA) tagged Kv11.1-1A and Kv11.1-3.1 constructs were generated via polymerase chain reaction and cloned into a genomic TTAA site targeting PiggyBac® pHULK® IRES-Serrano RFP® vector, also containing CMV-PAC in the transposed region (DNA2.0, Menlo Park, CA) Constructs were verified by bi-directional Sanger sequencing. Constructs were stably transfected with Lipofectamine 3000® (Life Technologies, Grand Island, NY) into HEK 293 cells. Transfected cells were then incubated for 48 hours at 37 °C in media containing 1 μg/mL puromycin (Sigma Aldrich, Saint Louis, MO). Single clones were selected by flow cytometry for red fluorescence and cultured until clonal lines were established. Expression was verified by western blot and whole-cell patch clamp. Scaled-up colonies were maintained in media containing 1μg/mL puromycin. HEK 293 cells were cultured at 37 °C (5% CO_2_) in Glutamax® Dulbecco’s modified Eagle’s Medium/Ham’s F-12 (Life Technologies, Grand Island, NY) supplemented with 1x Non-essential amino acids (Life Technologies, Grand Island, NY), 1x Penicillin/Streptomycin (Life Technologies, Grand Island, NY) and 10% fetal bovine serum (Sigma Aldrich, Saint Louis, MO). For pharmacological treatments, drugs were dissolved in DMSO and added to media at DMSO final concentrations < 0.2%. Cells were incubated in drug-containing media for 16–20 hours prior to assays.

### Electrophysiology

HEK 293 cells stably expressing hERG channels were dissociated and plated onto PDL-coated glass coverslips (Corning, Tewksbury, MA). Glass patch pipette electrodes were pulled using a PC-10® two-stage vertical puller (Narishige, Tokyo, Japan). Average pipette resistance was between 2–3.5MΩ when filled with an internal solution containing: 120 mM potassium gluconate, 5 mM EGTA, 10 mM HEPES, 20mM KCl, 1.5 mM Mg-ATP, at pH 7.3 with KOH. Plated cells were immersed in a perfused extracellular solution bath containing: 1 mM MgCl_2_, 1 mM CaCl_2_, 10 mM HEPES, 12.5 mM Glucose, 5 mM KCl, 130 mM NaCl, 0.1% dimethyl sulfoxide (DMSO), at pH 7.4 with NaOH. Liquid junction potential for these solutions was calculated to be −15 mV, which was not corrected for in experiments. Cells were voltage clamped in whole cell mode using an Axopatch® 200B amplifier (Molecular Devices, Sunnyvale, CA). Current signal was digitized at 5 kHz and filtered at 10 kHz and stored on an IBM-compatible PC interfaced with a NI USB-6221 analog-digital converter (National Instruments, Austin, TX).

### Voltage Protocols

All voltages protocols used are illustrated on or above the relevant panel. To induce both steady-state and tail currents, cells were held at a potential of −80 mV, depolarized to 0 mV for 5 s and then hyperpolarized to −120 mV for 3 s. To measure rates of deactivation, cells were first depolarized to 20 mV for 500 ms to completely activate the channels. Cells were then repolarized to voltages in the range −60 to −130 mV for 5 s and traces were fitted to biexponential functions as described in Heide *et al.*^9^.

### SDS-PAGE electrophoresis and Western Blot

Cells were washed with ice-cold PBS, harvested in ice-cold PBS, and pelleted by centrifugation at 500 g for 5 minutes. Cell pellets were resuspended with Radioimmunoprecipitation assay (RIPA) buffer (Sigma Aldrich, Saint Louis, MO) and rotated at 4 °C for 1 hour. Lysates were cleared by centrifugation at 16,000 g for 10 minutes and supernatants were analyzed for total protein concentration using a BCA kit (Pierce, Rockford, Illinois). 30 μg of total protein lysate was taken from each sample and fractionated on a 4–12% gradient Novex® Bis-Tris Bolt® SDS-PAGE gel via electrophoresis. Proteins were transferred onto 0.45 μm nitrocellulose membranes and incubated for 1hr in Odyssey® PBS blocking buffer (Li-Cor Biosciences, Lincoln, NE). Membranes were probed with anti-HA (1:5,000, GeneTex, Irvine, CA) and anti-NaK-ATPase (1:2,500, GeneTex, Irvine, CA) primary antibodies in Odyssey® PBS blocking buffer overnight at 4 °C. Anti-HA and anti-NaK-ATPase signals were detected using IRdye 800 donkey anti-rabbit (1:20,000, Li-Cor Biosciences, Lincoln, NE) and IRdye 680 donkey anti-mouse (1:20,000, Li-Cor Biosciences), respectively. The Li-Cor Odyssey® imaging system and software was used for antibody detection and quantification.

### Co-immunoprecipitation

Dynabeads Protein G® (Life Technologies, Grand Island, NY) were prepared according to the manufacturer’s instructions and incubated with HA.11 anti-HA antibody (BioLegend, San Diego, CA) for 30 minutes and then fixed with BS^3^ crosslinking reagent (ThermoFischer Scientific, Waltham, MA) to prevent antibody dissociation. Equal amounts of cell lysates from stably expressing Kv11.1 cell lines were prepared as before and then incubated with the prepared beads overnight at 4 °C. Beads were washed and proteins were eluted per the manufacturer’s instructions and visualized via SDS-PAGE. Membranes were probed with the antibodies described previously and anti-ubiquitin (1:1000, Abcam, Cambridge, MA). Antibody detection was performed as before.

### Fluorescence Microscopy

Stable HEK 293 cell lines expressing either the full-length (1A) or deletion Kv11.1 (3.1) channel variant were plated onto 24 well ibiTreat® plates (ibidi, Martinsried, Germany) and grown until 80–90% confluent. For sub-cellular co-localization studies, cells were fixed with 4% paraformaldehyde in PBS for 15 minutes. Cells were simultaneously permeabilized, blocked, and quenched for background fluorescence by incubation in a PBS solution containing 0.1% Triton X-100, 10% normal goat serum, and 0.75% glycine for 15 minutes. Cells were probed with primary antibodies: anti-extracellular loop hERG (1:1000, Sigma Aldrich, Saint Louis, MO), anti-calnexin (1:1000, Abcam, Cambridge, MA), anti-58K Golgi protein (1:200, Abcam), anti-20s proteasome β1 (1:200, Santa Cruz Biotechnology, Dallas, TX). For cell-surface labeling, fixation time was reduced to 10 minutes and paraformaldehyde concentration was reduced to 2% to reduce permeabilization. Cell surface labeling with anti-hERG antibody was performed as described above. Cell images were captured with the Operetta® high content imaging system and quantified with Columbus® image analysis suite (both PerkinElmer, Waltham, MA).

### FluxOR^®^ Thallium Flux Assay

Stably Kv11.1 channel expressing HEK 293 cells grown to 90% confluency were then plated onto PDL coated 384 well plates and allowed to grow 16–20 hours at 37 °C. FluxOR® loading buffer supplemented with 10 mM Red Dye #40 was added to each well and incubated for 45 minutes. Compounds were then added and incubated for 5 minutes. Stimulation buffer was added to plates in a FDSS7000 kinetic plate reader (Hamamatsu, Hamamatsu City, Japan) while channel activity was monitored. Channel activity was analyzed using the Hamamatsu FDSS software.

### Data Analysis and Statistics

Electrophysisology data analysis was performed using Axograph X® (Axograph, Berkley, CA) All data is presented as mean ± SEM unless specified. Unpaired T-tests, ANOVA, and subsequent Bonferroni corrections were completed using Prism (Graphpad, La Jolla, CA). Microscopy data was analyzed with linear mixed models with fixed effects for the treatment variables with a random intercept by replicate/well – hERG and RFP levels were log_2_-transformed prior to analysis. *P* values < 0.05 were accepted as significant.

## Results

### Kv11.1-3.1 is a poor expressing, trafficking deficient hERG channel

**Figure 1:**
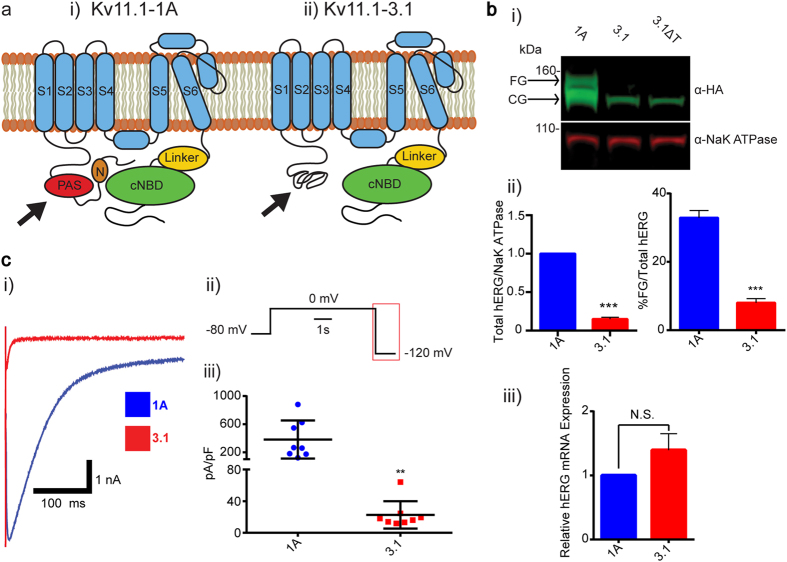
Kv11.1-3.1 is an N-terminally truncated hERG channel with trafficking, expression, and activity deficiencies. (**a**) Illustrative examples of Kv11.1-1A (i) and Kv11.1-3.1(ii) channel structure. Full length Kv11.1 channels contain N-terminal PAS domain and amphipathic helix N-cap, suggested to interact with each other and a C-terminal cyclic nucleotide biding domain. Kv11.1-3.1, being absent of a full PAS domain, likely presents an unstably folded N-terminal motif. (**b**) Sample Western blot (i) of whole cell lysates from stably transfected HEK 293 cells expressing recombinant HA-tagged Kv11.1-1A, Kv11.1-3.1, or Kv11.1-3.1 after 20 hour incubation at 28 °C (ΔT). Lysates were probed with anti-HA and anti-NaK ATPase primary antibodies subsequently incubated with fluorescent IR dyes for quantitative detection. Ladder markers indicated with respective sizes in kDa. Arrows indicate respective FG and CG bands representative of mature fully glycosylated and immature core glycosylated channel populations. Neither 3.1 nor cold-incubated 3.1 show obvious FG band expression, suggesting the 3.1 trafficking phenotype is heat-insensitive. (ii) Summary quantification of trafficking efficiency (FG/Total hERG, n = 3) and relative expression differences (Total hERG/NaK ATPase normalized to 1A expression, n = 3) of 1A and 3.1 channels. 3.1 showed significant decreases in both trafficking and steady-state expression. Note that while no FG signal is visible for Kv11.1-3.1, there is a photon count present above true background. (iii) Kv11.1-3.1 expressing cells show a non-significant increase in total hERG channel transcription compared to Kv11.1-1A expressing cells via qPCR (n = 3). (**c**) (i) Example whole-cell deactivation tail current voltage clamp traces from HEK 293 cells expressing 1A or 3.1 channels. (ii) Currents were recorded by holding cells at −80 mV, activating channels with a voltage step to 0 mV for 5s, followed by a hyperpolarizing step to −120 mV to induce tail currents. 3.1 displays rapid deactivation compared to 1A. (iii) Peak tail currents from 1A and 3.1 traces were normalized to cell capacitance (pA/pF, n = 8) and analyzed. 3.1 shows significantly reduce average peak tail current relative to 1A. All bar graph data presented as mean ± SEM (*P = <0.05, **P = <0.01, ***P = <0.001, ****P = <0.0001).

The HEK 293 cell overexpression model was used to determine steady-state expression levels, trafficking efficiency, and activity of Kv11.1-3.1, via electrophysiology, immunoblotting, and immunocytochemistry. With the intention of controlling for channel transcription and transfection efficiency, mRNA levels were evaluated by qPCR. Transcription levels from selected Kv11.1-1A and Kv11.1-3.1 cell lines did not vary significantly, although Kv11.1-3.1 displayed a non-significant increase relative to Kv11.1-1A transcription (P = 0.058) (Fig. 1b iii). Subsequent quantified data was not normalized to transcription levels due to these findings.

Channel activity was characterized via electrophysiology using whole-cell voltage clamp to determine efficacy in downstream HTS assays. Utilizing a test pulse protocol, by holding cells at −80 mV followed by depolarization at 0 mV and hyperpolarization at −120 mV (Fig. 1c ii), differences in tail current deactivation between Kv11.1-1A and Kv11.1-3.1 were evident (Fig. 1c i). Consistent peak tail current expression over 1 nA was considered acceptable for drug screening. The Kv11.1-1A line was quite robust in yielding large tail currents ( > 4 nA) as well as exhibiting a high fidelity of expression between cells. However, the Kv11.1-3.1 cell line did not produce a robust signal response in every cell, and it was uncommon to identify cells with tail current peaks over 1 nA. Cell membrane capacitance normalized peak tail currents from Kv11.1-3.1 expressing cells (22.66 ± 6.108 pA/pF, n = 8) were significantly lower (P = 0.0072) than currents recorded from Kv11.1-1A expressing cells (381.6 ± 95.82 pA/pF, n = 8) (Fig. 1c iii). Knowing that the expression rate and magnitude are critical for HTS methods^13^, it was concluded that even the best expressing Kv11.1-3.1 lines were insufficient for a drug discovery platform. Before investigating the nature of these deficits and approaches to improve channel activity, it was necessary to characterize expression and trafficking levels to better understand the cause of low channel activity.

Western blot analysis was used to quantify expression and trafficking, as reported by previous groups^27^. HEK 293 cells overexpressing Kv11.1 show two distinct bands via western blot: the mature fully glycosylated (FG) band and immature core glycosylated (CG) band. The core glycosylated band for Kv11.1-1A and Kv11.1-3.1 appears at 135kD and 125kD, respectively, while the FG band for each is approximately 20 kD higher (Fig. 1b i). These bands have been used previously as a heuristic diagnostic of channel trafficking^16^,^21^,^23^,^26^,^27^,^28^, as the lower band is representative of channel present in the ER, being subject to only one glycosylation event, while the FG band is indicative of successfully trafficked channel being glycosylated again in the Golgi^10^.

The overall steady-state expression was determined by summing the total photon signal from both FG and CG bands normalized by the NaK ATPase loading control count. It was discovered that basal Kv11.1-3.1 expression was only 15 ± 2% (P = 0.0008) of that to Kv11.1-1A levels on the same blot (Fig. 1b ii). Furthermore, by calculating trafficking expression as a function of FG photon count normalized by total hERG photon count (FG + CG) it was found that Kv11.1-1A and Kv11.1-3.1 cells exhibited a significantly different (P = 0.0002) trafficking efficiency of 32 ± 2% (n = 3) and 8 ± 1% (n = 3) respectively (Fig. 1b ii).

**Figure 2:**
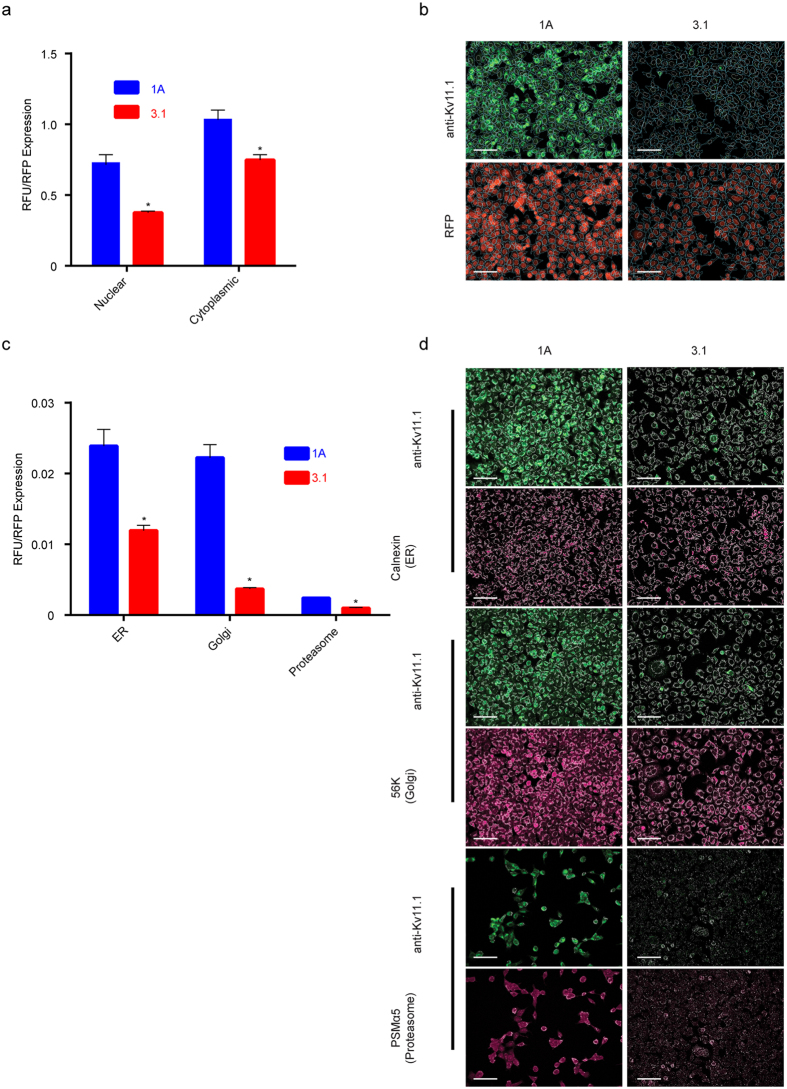
Single cell measurements of Kv11.1-1A and Kv11.1-3.1 show reduced expression across sub-cellular compartments. (**a**) Total Kv11.1-1A and Kv11.1-3.1 signal (RFU) was measured in each cell and normalized to the RFP signal from the same cells. Note the reduced signal in 3.1 from both nucleus and cytoplasm (n = 3 unique experiments). (**b**) Representative fluorescence micrographs show both fluorescence signal and overlay after sub-cellular image segmentation. (**c**) RFP-normalized signal from indicated sub-cellular compartments (n = 3 unique experiments). (**d**) Fluorescence micrographs demonstrate localized signal from Kv11.1 and the organelle-specific masks used for sub-cellular localization. Colocalization and segmentation data used is from same experiment in Fig. 5. Scale bars = 100μm. All bar graph data presented as mean ± SEM (*P = <0.05).

To establish the sub-cellular localization of Kv11.1-1A and −3.1, we performed co-localization studies using antibodies against Kv11.1 and control sub-cellular markers (Fig. 2). Kv11.1 signal was measured in the nucleus and cytoplasm using DAPI as a differential counterstain for the nucleus and cytoplasm (Fig. 2a). To normalize the anti-Kv11.1 antibody fluorescence in each cell, we also measured signal from the red fluorescent protein (RFP) that is expressed from an internal ribosome entry site (IRES). Characteristic fluorescence micrographs demonstrate the localization of both transgenes to the cytoplasm (Fig. 2b). Total Kv11.1 fluorescence was measured in domains defined by the expression of the control organelle markers calnexin (ER), 58k (Golgi), and alpha 5 (proteasome) (Fig. 2c,d). The fluorescence signal from Kv11.1-3.1 was significantly lower in all measured domains.

### Kv11.1-3.1 expression and trafficking is rescued through proteasome inhibition

It has been demonstrated that many Kv11.1-1A LQT2 mutant trafficking defects are responsive to overnight low temperature incubation, while some are completely unaffected^25^. Upon incubation of Kv11.1-3.1 expressing HEK 293 cells at 28 °C for 16–20 hours, no FG band rescue was seen by Western (Fig. 1b i). Knowing that Kv11.1-3.1 trafficking in HEK 293 cells was non-responsive to cold incubation, pharmacological rescue agents were then investigated using the same phenotypic Western blot screening.

**Figure 3:**
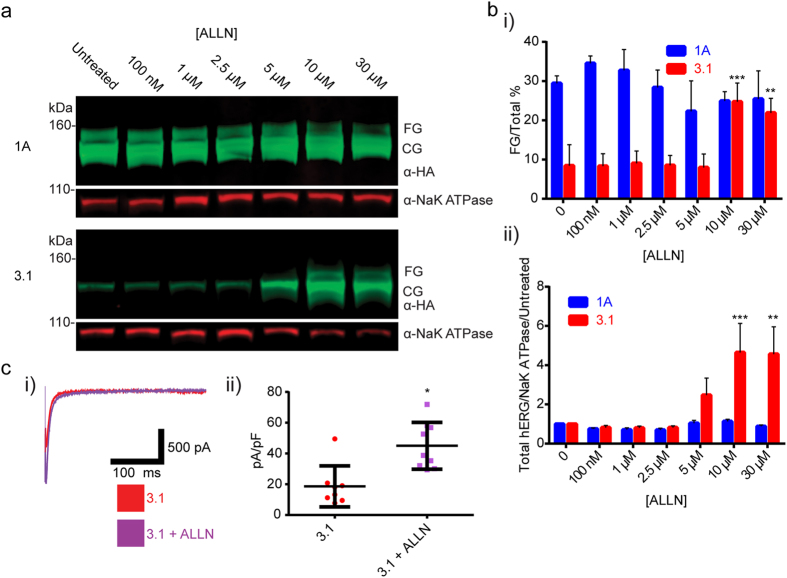
Kv11.1-3.1 expression, trafficking, and peak tail currents are significantly increased via pharmacological rescue with ALLN. (**a**) Example Western blot analysis of cells expressing 1A or 3.1 treated with the calpain/lysosomal cathepsin/proteasome inhibitor ALLN. ALLN treatment of 3.1 expressing cells resulted in presence of the FG band and increased CG band intensity while only increasing 1A CG expression. (**b**) Quantitation summary of Western analysis shows significant increases of i) 3.1 trafficking and ii) total hERG expression upon ALLN treatment (n = 3). No significant effects were seen in 1A expressing cells. (**c**) i) Representative whole-cell voltage clamp tail current traces for ALLN treated 3.1 cells. ii) Peak tail currents were normalized to cell capacitance (n = 8) and analyzed. ALLN treatment was found to significantly increase normalized peak tail currents compared to untreated cells (*P = <0.05). All bar graph data presented as mean ± SEM (*P = <0.05, **P = <0.01, ***P = <0.001).

A library of small molecules, that were either reported in the literature, or that were preliminarily identified for hERG activity, ERAD/UPR modulation, protease activity, or activity in other germane processes, was created^23^,^29^,^30^,^31^,^32^,^33^,^34^,^35^. Initially, it was found that the 26s proteasome inhibitor Mg132^36^ boosted CG expression levels, but was too cytotoxic for reproducible use (data not reported). Likewise, it was shown that the calpain/cathepsin/proteasome inhibitor N-[N-(N-acetyl-L-leucyl)-L-leucyl]-L-norlecucine (ALLN)^22^,^37^ greatly increased Kv11.1-3.1 CG expression. (Fig. 3a) Furthermore, ALLN was the only compound to show the clear presence of a Kv11.1-3.1 FG band after treatment (Fig 3a). Upon quantitative western analysis, optimal concentrations of ALLN rescued trafficking efficiency (25 ± 3%, n = 3, P = 0.0006) to similar levels seen in untreated Kv11.1-1A cells (Fig. 3b i) and increased overall channel expression by over 4-fold (Fig. 3b ii, P = 0.001). Whole-cell voltage clamp test pulses evoked larger peak tail currents for ALLN treated cells expressing Kv11.1-3.1, than for untreated controls (Fig. 3c i). Cell membrane capacitance normalized tail current peaks were found to be significantly greater (P = 0.0472) in the ALLN treated cell groups (45.02 ± 5.079 pA/pF, n = 9) compared to Kv11.1-3.1 controls (18.65 ± 4.714 pA/pF, n = 8) (Fig. 3c ii). To understand if this effect was selective for the Kv11.1-3.1 specific deficiencies, Kv11.1-1A expressing cells were treated with ALLN in a similar manner. Neither overall Kv11.1-1A expression nor trafficking efficiency was significantly altered with ALLN treatment (Fig. 3a,b).

**Figure 4:**
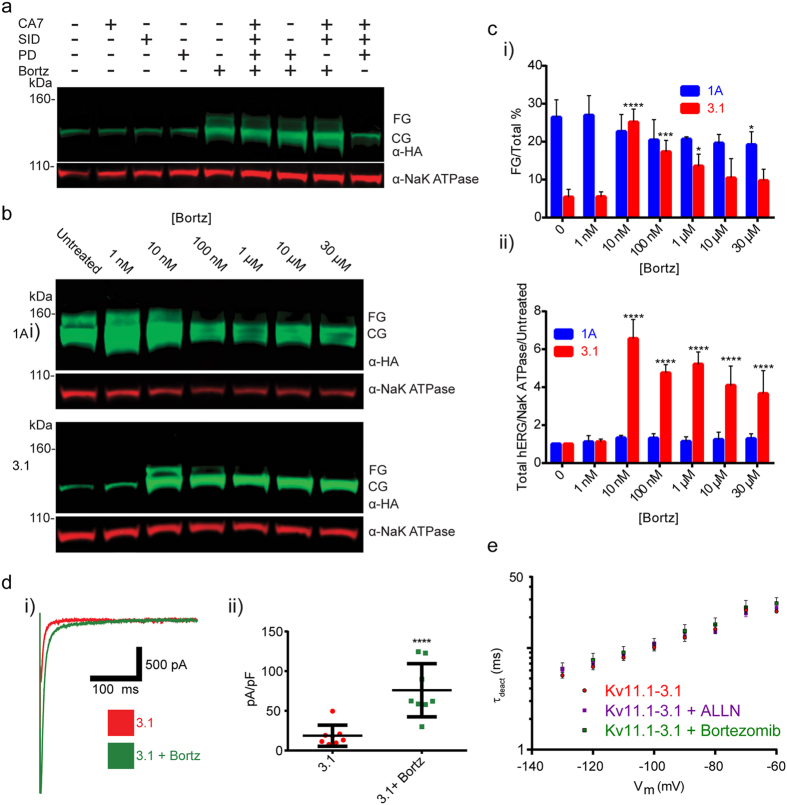
Proteasome inhibition is sufficient for Kv11.1-3.1 rescue via bortezomib. (**a**) Example blot of 3.1 expressing cells treated with either selective calpain I/II inhibitor PD 150606, lysosomal cathepsin B/L inhibitors CA 074/SID 26681509, 26s proteasome inhibitor bortezomib, or various cocktails in order to determine ALLN mechanism of rescue. Only samples treated with bortezomib, or a cocktail consisting thereof, resulted in the display of a FG band. (**b**) Example blot of cells expressing 1A or 3.1 treated with bortezomib. Treatment of 3.1 expressing cells with bortezomib results in presence of the FG band and increased CG band intensity while only increasing 1A CG expression. (**c**) Quantitation summary of Western analysis shows significant increases of i) 3.1 trafficking and ii) total hERG expression upon bortezomib treatment(n = 3). (**d**) i) Representative whole-cell voltage clamp tail current traces for bortezomib treated 3.1 cells. ii) Peak tail currents were normalized to cell capacitance (n = 8) and analyzed. Bortezomib treatment was found to significantly increase normalized peak tail currents compared to untreated cells (****P = <0.0001). Note data for untreated 3.1 peak tail currents is from the same experiments in Fig. 3c ii. (**e**) Pharmacological rescue conditions were tested for alterations to channel kinetics according to deactivation rate voltage protocol. i) Extrapolated τ_deact_ values for pharmacologically treated cells were not shown to be significantly different from those of controls, thus rescue compounds had no effect on Kv11.1-3.1 deactivation rates (n = 3). All bar graph data presented as mean ± SEM (*P = <0.05, ***P = <0.001, ****P = <0.0001).

Although these findings supported ALLN as a good candidate for a rescue agent, ALLN contains a reactive aldehyde species and exhibits polypharmacology across multiple proteases^24^,^38^. Consequently it was necessary to investigate the mechanism of rescue. By comparing τ for deactivation of ALLN treated Kv11.1-3.1 expressing cells and controls (Fig. 4e), it was demonstrated that no significant difference was seen in the primary Kv11.1-3.1/Kv11.1-1A-differentiating deactivation kinetics, thus rescue was not likely due to channel structure modification. In order to deconvolute the ALLN polypharmacology for principle pharmacodynamic contributions, Kv11.1-3.1 expressing cells were treated with small molecules selective for cathepsins B,L, calpains I,II and the 26s proteasome, the multiple ALLN targets. The calpain I/II inhibitor PD 150606, the cathepsin B inhibitor CA 078, the cathepsin L inhibitor SID 26681509, and the 26s proteasome inhibitor bortezomib were chosen^39^,^40^,^41^,^42^. It was also anticipated that the rescue activity of ALLN might arise from the specific polypharmacological profile, so various cocktails of these inhibitors were formulated for screening. Western blot analysis from these treated cells (Fig. 4a) yielded that only bortezomib was effective, hence 26s proteasome inhibition was responsible for Kv11.1-3.1 expression and trafficking increases.

Bortezomib was titrated for optimal rescue attributes and identical concentrations were tested in the Kv11.1-1A cell line (Fig. 4b). At 10 nM, bortezomib was found to rescue Kv11.1-3.1 channel trafficking (25 ± 2%, n = 4, P = <0.0001) (Fig. 4c i), increase overall Kv11.1-3.1 channel expression by an average of 6 fold (Fig. 4c ii, P = <0.0001), and increase peak tail currents (Fig. 4d i). The increase in cell membrane capacitance normalized peak tail currents (75.93 ± 11.84 pA/pF, n = 8) was found to be greater than that of untreated Kv11.1-3.1 expressing cells (Fig. 4d ii, P = <0.0001). Bortezomib treatment had no effect on Kv11.1-3.1 deactivation rate kinetics (Fig. 4e).

**Figure 5:**
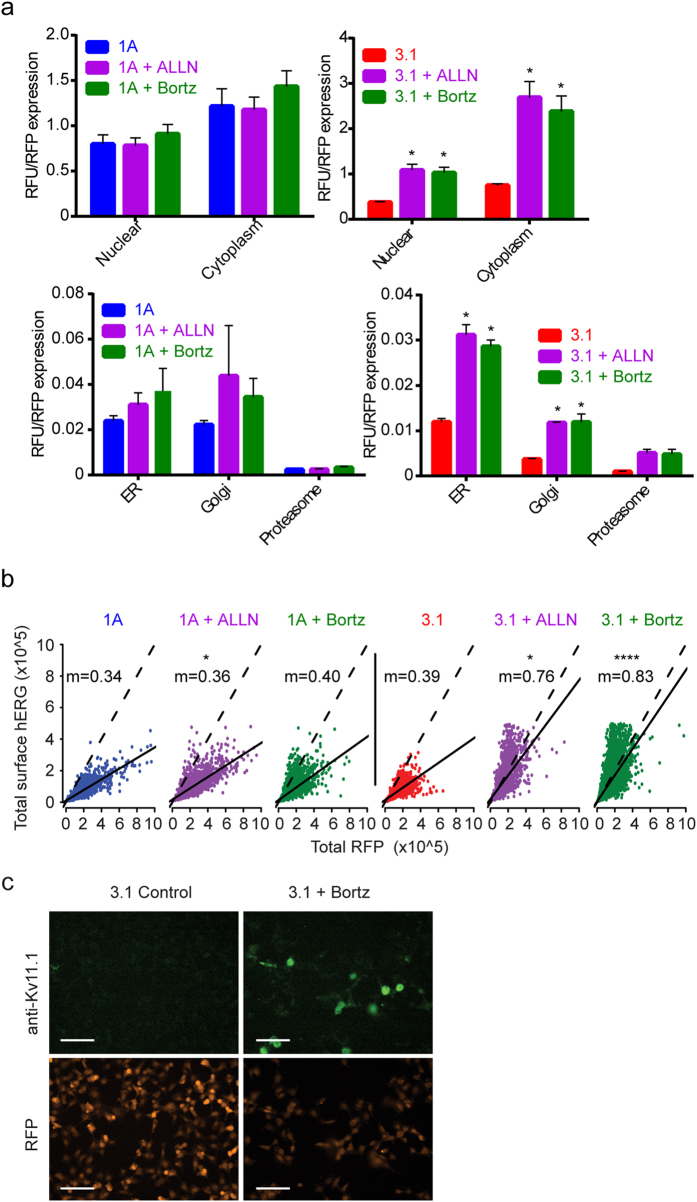
Proteasome inhibitor treated cells show Kv11.1-3.1 rescue across sub-cellular compartments and cell surface. (**a**) RFP-normalized fluorescence of Kv11.1 1A and 3.1. Note the differential elevation of 3.1 compared to 1A after the same treatments (n = 3). (**b**) Scatter plot of RFP vs surface Kv11.1 in single cells, n > 3000 measurements per condition (n = 3 unique experiments). m = slope of the linear regression (solid line). The dotted lines have slope m = 1 for reference. (**c**) Immunofluorescence micrographs of representative fields showing specific surface Kv11.1-3.1. Scale bars = 100 μm. All bar graph data presented as mean ± SEM (*P = <0.05, ****P = <0.0001).

Quantitative immunofluorescence of Kv11.1 1A and Kv11.1-3.1 supports the conclusion from electrophysiological studies that low Kv11.1-3.1 levels are a consequence of a proteasomal mechanism (Fig. 5). Although modest elevation of Kv11.1 1A was detected when cultured in the presence of ALLN and bortezomib, a nearly 3-fold elevation of steady-state protein level was detected in the Kv11.1-3.1 variant when treated with ALLN or bortezomib (Fig. 5a). When analyzed at subcellular segmentation and colocalization domains, both ALLN and bortezomib significantly increased Kv11.1-3.1 expression in the cytoplasm and nuclear regions along with the ER and Golgi. However, the increases in Kv11.1-3.1 proteasome domain expression were not found to be significant. Increased electrophysiological activity of Kv11.1-3.1 in the presence of bortezomib should involve increased localization of this channel to the cell surface. Using an antibody that recognizes an extra-cellular loop found in both Kv11.1-1A and Kv11.1-3.1, we performed immunocytochemistry with a brief fixation protocol that maintains the plasma membrane integrity (Fig. 5b). To normalize the anti-Kv11.1 antibody fluorescence in each cell, we also measured signal from the red fluorescent protein (RFP) that is expressed from an internal ribosome entry site (IRES). Thus, the mRNAs for both Kv11.1 and RFP are regulated by a single common promoter, while the relative stability of each protein is a consequence of post-transcriptional mechanisms. The ratio of Kv11.1-1A/RFP (m = 0.34) is minimally altered by ALLN (m = 0.36, P = 0.029) and bortezomib (m = 0.40, n.s.) as shown by the similar linear regression slopes (Fig. 5b). In contrast, bortezomib and ALLN increase this ratio (b = 0.39) by a factor of 2 in the Kv11.1-3.1 variant (m = 0.75, P = 0.028 and m = 0.83, P = <0.0001 respectively) (Fig. 5b), demonstrating specific elevation of the Kv11.1-3.1 variant surface abundance with these small molecules. Representative fluorescence micrographs of Kv11.1 3.1 alone and in the presence of ALLN demonstrate the dramatic specific elevation of Kv11.1 3.1 (Fig. 5c). Note the increase in Kv11.1 signal without change of RFP in the Kv11.1-3.1 variant only in the presence of ALLN or bortezomib. These data provide the third independent measurement of Kv11.1 3.1 demonstrating increased protein stability in the presence of proteasome inhibitors.

**Figure 6:**
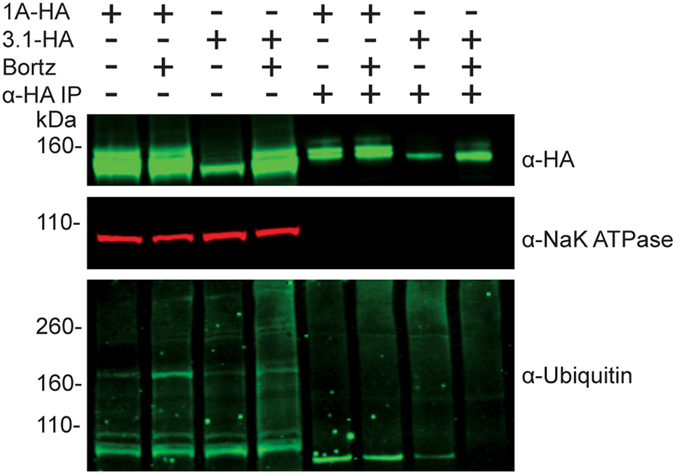
Kv11.1-1A and Kv11.1-3.1 show different channel ubiquitinylated changes in response to bortezomib. HA tagged hERG channels were immunoprecipated from stably expressing hERG cell lysates that had been incubated with bortezomib or DMSO for 16 hours. Cell lysates and IP elutions were probed for HA tag, NaK ATPase, and ubiquitin. Cell lysates show increases in total ubiquitin with bortezomib treatment and both 1A and 3.1 channels show poly-ubiquitinylation. Immunoprecipitated 1A channels show increase in ubiquitnylation accumulation in bortezomib treatments. Immunoprecipitated 3.1 channels from bortezomib treated cells show less accumulation than DMSO incubated cells.

While it is known Kv11.1-1A channels are ubiquitinylated in HEK 293 cells prior to proteasome degradation^43^, no such mechanism has been shown for Kv11.1-3.1 channels, critical in exploring potential proteasome-dependent regulation of Kv11.1-3.1 channels. Immunoprecipitation of Kv11.1-1A and Kv11.1-3.1 channels was performed with and without pharmacological recue conditions with bortezomib (Fig. 6). Immunoprecipitated channels lacked lysate loading control bands, and mouse IGG only samples (data not shown) did not yield signal, suggesting the IP was perfomed without residual non-specific proteins. Immunoprecipitation of the respective channels showed similar hERG signals as before, however the CG/FG band locates were slighty shifted up, likely due to different loading buffer conditions from the Protein A bead dissociation. Cell lysates from the respective cell lines showed increases in ubiquitin signal with bortezomib treatment. Immunoprecipitated 1A channels showed ubiquitinylation and accumulation of ubiquitin signal upon bortezomib treatment. Immunoprecipitated Kv11.1-3.1 channels showed ubiquitinylation, however, a decrease in ubiquitin signal upon bortezomib treatment.

### Rescued Kv11.1-3.1 activity is suitable for High Throughput Screening

**Figure 7:**
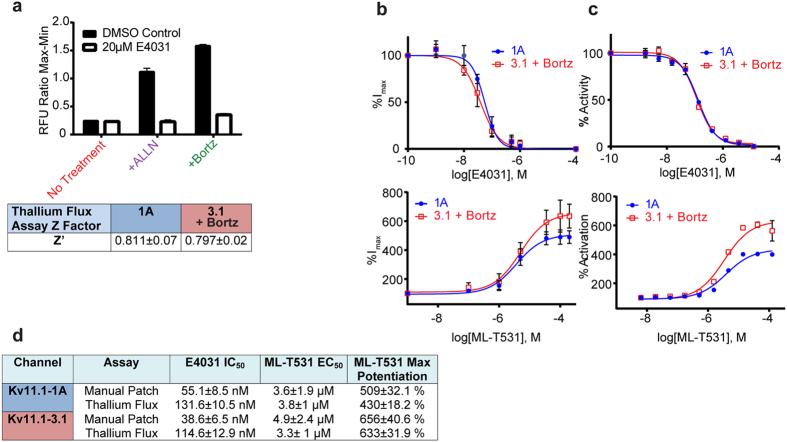
Bortezomib treatment of Kv11.1-3.1 expressing cells produces sufficient currents for dose-response analysis of hERG inhibitors and activators via whole cell voltage clamp and high throughput fluorescence methods. (**a**) 3.1 expressing cells were treated with ALLN or bortezomib as before in 384 well assay plates. Plates were incubated the next day with assay loading buffer for 45 minutes, and then incubated with either E4031 or DMSO for 5 minutes prior to addition of stimulation buffer and subsequent signal detection. Comparison of pharmacologically treated 3.1 expressing cells and controls shows that both ALLN and bortezomib tremendously increase signal to background window in the thallium flux assay (n = 16). Z’ scores for the thallium flux assay with either 1A cells or 3.1 cells treated with bortezomib are well above the 0.5 threshold across multiple trials (n = 3). (**b**) Dose-response of peak tail currents to either E4031 (n = 4) or ML-T531 (1A n = 3, 3.1 n = 6) titration in whole-cell voltage clamp of cells expressing either 1A or 3.1 treated with bortezomib. (**c**) Dose-response of peak tail currents to either E4031 (n = 4) or ML-T531 (1A n = 3, 3.1 n = 6) titration in thallium flux assay of cells expressing either 1A or 3.1 treated with bortezomib. (**d**) Table summary of compound potencies and activator potentiation data. E4031 potencies decrease in thallium flux assay compared to voltage-clamp by less than 3 fold. ML-T531 exhibits higher max potentiation in 3.1 channels than 1A. All graph and table data presented as mean ± SEM.

To determine if these rescue strategies could be successfully employed for a HTS assay, Kv11.1-3.1 expressing cells were treated as in the Western assays, and plated in multi-well plates in preparation for a potassium channel flux assay based on a thallium-sensitive dye^14^. ALLN and bortezomib treated cells both yielded significant signal improvement compared to untreated cells in the 384 well thallium flux assay with low variations between wells, both showing roughly 4 fold signal to background ratios (Fig. 7a). Due to its lower effective concentration and repeated superiority to ALLN in assays for expression and trafficking, bortezomib was chosen as the rescue agent in subsequent HTS assays.

Using bortezomib treated Kv11.1-3.1 and untreated Kv11.1-1A cells, multiple assay plates were run in the thallium flux assay with the known Kv11.1 inhibitor E4031 and DMSO as controls. Repeated assay plates generated z’ scores of 0.811 ± 0.07 for Kv11.1-1A cells without proteasome inhibitor and 0.797 ± 0.02 for treated Kv11.1-3.1 cells (Fig. 7a). Dose-response curves were generated (Fig. 7c) for E4031 inhibition, and a known hERG activator, ML-T531^35^. Additionally, these small molecules were titrated with treated Kv11.1-3.1 and Kv11.1-1A cells in the same voltage-clamp assay used to determine capacitance normalized tail current (Fig. 7b) for assay comparison. E4031 had uniformly 3-fold less potency against both channels (Fig. 7d) in the thallium flux assay as compared to voltage clamp, showing no significant selectivity between channels. EC_50_ values for ML-T531 were similarly identical for Kv11.1-1A and Kv11.1-3.1 in these assays, near 4μM. However, a higher maximum potentiation effect was seen for Kv11.1-3.1 as compared to Kv11.1-1A. EC_50_ values were also conserved across assay platforms, in contrast to E4031 IC_50_ values (Fig. 7d).

## Discussion

In this study it was confirmed that Kv11.1-3.1 is a poor-expressing channel and that it displays impaired trafficking and channel activity (Fig. 1). This data reflects general observations made concerning Kv11.1-3.1 expression in HEK cells via Western analysis^8^,^9^; however, this is the first time these characteristics have been quantified, analyzed, and also reflected in fluorescent microscopy (Fig. 2) to our knowledge. Of particular interest are the general subcellular localization analyses; these demonstrate Kv11.1-1A is expressed over 6 fold higher in the Golgi, 2 fold higher in the ER, and 2 fold higher in the proteasome domains, as compared to Kv11.1-3.1. While 1A expression in the Golgi and ER is similar, there is a much greater ratio of Kv11.1-3.1 in the ER/Golgi, suggesting a differential kinetic distribution and thus supporting the trafficking hypothesis. Using the same methods, it was found that inhibition of proteasome activity increased steady-state expression, plasma membrane trafficking, and subsequent peak current of Kv11.1-3.1. (Figs. 3, 4, 5) These findings were applied to the fluorescent 384 well plate thallium flux assay for potassium channel activation, and it was found that rescue via proteasome inhibition resulted in a robust response, amenable for HTS and subsequent drug discovery studies. In general, the discoveries outlined in this report suggest two principal implications: the role of ERAD and UPR in Kv11.1-3.1 regulation and a potential translational component.

Proteasome involvement is known in hERG trafficking and degradation^10^,^26^, but is not often labeled as the key factor in these pathways. Nonspecific ALLN treatment has previosuly shown rescue of general hERG 1A expression when coexpressed heterogenously with a dominant negative LQT2 mutant, although the effects on trafficking are not clear^37^. Although proteasomal inhibition via lactacystin and Mg132 treatment has been shown previously to increase hERG expression for some LQT2 mutants^26^, trafficking was unable to be rescued suggesting a different limiting factor. The majority of hERG trafficking studies target LQT2 clinically relevant missense mutations and PAS truncations^10^. These cause channel retention in different compartments of the endoplasmic reticulum^34^, which may vary on the mutation despite similar CG channel expression. Similar studies include the more severely truncated variant Kv11.1-1B, which results in analogous observations due to the expression of RXR retention motifs^44^. Successful rescue strategies for trafficking deficient Kv11.1-1 channels include pharmacological chaperones, incubation at reduced temperature, and expression of heterotetramers with WT Kv11.1-1A. These rescue techniques either assist in stabilizing channels or disrupt chaperone mediated quality control mechanisms^10^,^21^,^23^,^24^,^25^,^44^.

In contrast, we hypothesize that Kv11.1-3-1’s unstably folded N-terminal region, caused by alternative transcription, results in a unique kinetic distribution amongst ER quality control check points and possibly different chaperone interactions. Due to insensitivity to other rescue strategies (Fig. 1), the robust response from bortezomib and ALLN (Figs 3, 4, 5), and lack of specific 3.1 ubiquitinylation upon proteasome inhibition (Fig. 6), it is possible that Kv11.1-3.1 channels are more rapidly degraded by the proteasome than other defective hERG channels. Therefore, it may be improper to describe Kv11.1-3.1 as a truly trafficking deficient channel. In fact, there may be utility in comparing Kv11.1-3.1 to the previously reported Δ2–135 truncated hERG channel^8^. Despite absence of the PAS domain and N-cap, Δ2–135 has increased trafficking and similar stability compared to Kv11.1-1A. In a sense, Kv11.1-3.1 may be seen as analogous to Δ2–135 with the addition of a small misfolded region causing immediate targeting to the proteasome. It may also be worthwhile to examine LQT2 mutants, resistant to typical methods of rescue, under proteasome inhibition in HEK cells.

Continued exploration of Kv11.1-3.1’s trafficking mechanisms to the proteasome in brain and other cell lines may be of interest to discover new therapeutic targets that increase Kv11.1-3.1 retention and degradation in patients with abnormally high Kv11.1-3.1 expression. Because Kv11.1-3.1 is expressed in healthy controls^5^, increased Kv11.1-3.1 expression alone is not sufficient for a disease state. Considering previous work that has shown UPR to be down regulated in some groups of schizophrenia patients^45^,^46^, it may be of value to investigate the effect of these two possible risk factors. As this study suggests ERAD and UPR play a large role in the expression and trafficking of Kv11.1-3.1, one may imagine a feed-forward effect in a down-regulated UPR patient.

In addition to basic channel regulation, these results provided insight into the abilities of using Kv11.1-3.1 for drug discovery. Through proteasome inhibition, the Kv11.1-3.1 signal was improved and resulted in gratifying enhancement of Z’ values comparable to Kv11.1-1A. The two tool compounds, E4031 and ML-T531, displayed fidelity between the voltage-clamp and fluorescent thallium flux assay (Fig. 5d). Generally, IC_50_/EC_50_ values were consistent between assays, although the thallium flux yielded 3 fold lower potencies for E4031 in both Kv11.1-1A and Kv11.1-3.1 expressing cells. Nevertheless, thallium flux data is known to correlate well (albeit not perfectly) to voltage-clamp, and these results are consistent with previous studies comparing E4031 potencies between the two assays^14^.

A principal goal of this assay is to determine selectivity of molecules for the Kv11.1-3.1 channel in order to avoid future cardiac toxicity. Neither the hERG inhibitor E4031 nor activator ML-T531 showed selectivity between Kv11.1-1A and Kv11.1-3.1. However, it was seen that ML-T531 significantly potentiated Kv11.1-3.1 current more so than Kv11.1-1A. While it is unclear as to why this is observed, it should be noted that the EC_50_ between channels was essentially identical. Therefore, the differences in potentiation increase may be attributed to intrinsic differences amongst channel fluxes and it may be considered that even nonselective compounds have a small “built-in” selectivity for Kv11.1-3.1 as compared to Kv11.1-1A.

In this study vital infrastructure has been established to interrogate Kv11.1-3.1 as a drug discovery target for new schizophrenia therapeutics through simultaneously unveiling a novel mechanism for hERG channel trafficking deficiency via rapid proteasomal degradation. While efforts to establish a pipeline for this novel cognitive target are in preliminary stages, these findings present an opportunity to discover other interacting druggable targets and create the tools necessary for identifying selective molecules.

## Additional Information

**How to cite this article**: Calcaterra, N. E. *et al.* Schizophrenia-Associated hERG channel Kv11.1-3.1 Exhibits a Unique Trafficking Deficit that is Rescued Through Proteasome Inhibition for High Throughput Screening. *Sci. Rep.* **6**, 19976; doi: 10.1038/srep19976 (2016).

## Supplementary Material

Supplementary Information

## References

[b1] Schubert C. R., Xi H. S., Wendland J. R. & O’Donnell P. Translating human genetics into novel treatment targets for schizophrenia. Neuron 84, 537–541 (2014). 25442931 10.1016/j.neuron.2014.10.037

[b2] Network & Pathway Analysis Subgroup of Psychiatric Genomics, C. Psychiatric genome-wide association study analyses implicate neuronal, immune and histone pathways. Nat. Neurosci. 18, 199–209 (2015). 25599223 10.1038/nn.3922 PMC4378867

[b3] Schizophrenia Working Group of the Psychiatric Genomics, C. Biological insights from 108 schizophrenia-associated genetic loci. Nature 511, 421–427 (2014). 25056061 10.1038/nature13595 PMC4112379

[b4] Harrison P. J. The current and potential impact of genetics and genomics on neuropsychopharmacology. Eur. Neuropsychopharmacol. 25, 671–681 (2015). 23528807 10.1016/j.euroneuro.2013.02.005

[b5] Huffaker S. J. *et al.* A primate-specific, brain isoform of KCNH2 affects cortical physiology, cognition, neuronal repolarization and risk of schizophrenia. Nat. Med. 15, 509–518 (2009). 19412172 10.1038/nm.1962 PMC2756110

[b6] Apud J. A., Zhang F., Decot H., Bigos K. L. & Weinberger D. R. Genetic variation in KCNH2 associated with expression in the brain of a unique hERG isoform modulates treatment response in patients with schizophrenia. Am. J. Psychiatry 169, 725–734 (2012). 22706279 10.1176/appi.ajp.2012.11081214

[b7] Hashimoto R. *et al.* The KCNH2 gene is associated with neurocognition and the risk of schizophrenia. World J. Biol. Psychiatry 14, 114–120 (2013). 21936766 10.3109/15622975.2011.604350

[b8] Ke Y., Hunter M. J., Ng C. A., Perry M. D. & Vandenberg J. I. Role of the cytoplasmic N-terminal Cap and Per-Arnt-Sim (PAS) domain in trafficking and stabilization of Kv11.1 channels. J. Biol. Chem. 289, 13782–13791 (2014). 24695734 10.1074/jbc.M113.531277 PMC4022852

[b9] Heide J., Mann S. A. & Vandenberg J. I. The schizophrenia-associated Kv11.1-3.1 isoform results in reduced current accumulation during repetitive brief depolarizations. PLoS One 7, e45624 (2012). 23029143 10.1371/journal.pone.0045624 PMC3454411

[b10] Vandenberg J. I. *et al.* hERG K(+) channels: structure, function, and clinical significance. Physiol. Rev. 92, 1393–1478 (2012). 22988594 10.1152/physrev.00036.2011

[b11] Johnson J. N. *et al.* Identification of a possible pathogenic link between congenital long QT syndrome and epilepsy. Neurology 72, 224–231 (2009). 19038855 10.1212/01.wnl.0000335760.02995.ca PMC2677528

[b12] Ji H. F. *et al.* Functional characterization of ether-a-go-go-related gene potassium channels in midbrain dopamine neurons - implications for a role in depolarization block. Eur. J. Neurosci. 36, 2906–2916 (2012). 22780096 10.1111/j.1460-9568.2012.08190.x PMC4042402

[b13] Farre C. & Fertig N. HTS techniques for patch clamp-based ion channel screening - advances and economy. Expert Opin Drug Discov 7, 515–524 (2012). 22507077 10.1517/17460441.2012.682056

[b14] Titus S. A. *et al.* A new homogeneous high-throughput screening assay for profiling compound activity on the human ether-a-go-go-related gene channel. Anal. Biochem. 394, 30–38 (2009). 19583963 10.1016/j.ab.2009.07.003 PMC2766802

[b15] Gianulis E. C. & Trudeau M. C. Rescue of aberrant gating by a genetically encoded PAS (Per-Arnt-Sim) domain in several long QT syndrome mutant human ether-a-go-go-related gene potassium channels. J. Biol. Chem. 286, 22160–22169 (2011). 21536673 10.1074/jbc.M110.205948 PMC3121360

[b16] Ke Y. *et al.* Trafficking defects in PAS domain mutant Kv11.1 channels: roles of reduced domain stability and altered domain-domain interactions. Biochem. J. 454, 69–77 (2013). 23721480 10.1042/BJ20130328

[b17] Anderson C. L. *et al.* Large-scale mutational analysis of Kv11.1 reveals molecular insights into type 2 long QT syndrome. Nat Commun 5 (2014). 10.1038/ncomms6535 PMC4243539 25417810

[b18] Gianulis E. C., Liu Q. & Trudeau M. C. Direct interaction of eag domains and cyclic nucleotide-binding homology domains regulate deactivation gating in hERG channels. J. Gen. Physiol. 142, 351–366 (2013). 24043860 10.1085/jgp.201310995 PMC3787778

[b19] Gustina A. S. & Trudeau M. C. The eag domain regulates hERG channel inactivation gating via a direct interaction. J. Gen. Physiol. 141, 229–241 (2013). 23319729 10.1085/jgp.201210870 PMC3557309

[b20] Haitin Y., Carlson A. E. & Zagotta W. N. The structural mechanism of KCNH-channel regulation by the eag domain. Nature 501, 444–448 (2013). 23975098 10.1038/nature12487 PMC3910112

[b21] Gong Q. M., Anderson C. L., January C. T. & Zhou Z. F. Pharmacological rescue of trafficking defective HERG channels formed by coassembly of wild type and long QT mutant N470D subunits. Biophys. J. 82, 253a–254a (2002). 10.1152/ajpheart.00052.2004 15072950

[b22] Mehta A. *et al.* Re-trafficking of hERG reverses long QT syndrome 2 phenotype in human iPS-derived cardiomyocytes. Cardiovasc. Res. 102, 497–506 (2014). 24623279 10.1093/cvr/cvu060

[b23] Smith J. L. *et al.* Pharmacological correction of long QT-linked mutations in KCNH2 (hERG) increases the trafficking of Kv11.1 channels stored in the transitional endoplasmic reticulum. Am. J. Physiol. Cell Physiol. 305, C919–930 (2013). 23864605 10.1152/ajpcell.00406.2012 PMC4042535

[b24] Zhang K. P., Yang B. F. & Li B. X. Translational toxicology and rescue strategies of the hERG channel dysfunction: biochemical and molecular mechanistic aspects. Acta Pharmacol. Sin. 35, 1473–1484 (2014). 25418379 10.1038/aps.2014.101 PMC4261120

[b25] Zhou Z., Gong Q. & January C. T. Correction of defective protein trafficking of a mutant HERG potassium channel in human long QT syndrome. Pharmacological and temperature effects. J. Biol. Chem. 274, 31123–31126 (1999). 10531299 10.1074/jbc.274.44.31123

[b26] Mihic A., Chauhan V. S., Gao X., Oudit G. Y. & Tsushima R. G. Trafficking defect and proteasomal degradation contribute to the phenotype of a novel KCNH2 long QT syndrome mutation. PLoS One 6, e18273 (2011). 21483829 10.1371/journal.pone.0018273 PMC3069070

[b27] Walker V. E. *et al.* Hsp40 chaperones promote degradation of the HERG potassium channel. J. Biol. Chem. 285, 3319–3329 (2010). 19940115 10.1074/jbc.M109.024000 PMC2823420

[b28] Thomas D., Kiehn J., Katus H. A. & Karle C. A. Defective protein trafficking in hERG-associated hereditary long QT syndrome (LQT2): molecular mechanisms and restoration of intracellular protein processing. Cardiovasc. Res. 60, 235–241 (2003). 14613852 10.1016/j.cardiores.2003.08.002

[b29] Zhou Z. F., Gong Q. M. & January C. T. Correction of defective protein trafficking of a mutant HERG potassium channel in human long QT syndrome - Pharmacological and temperature effects. J. Biol. Chem. 274, 31123–31126 (1999). 10531299 10.1074/jbc.274.44.31123

[b30] Zhou J. *et al.* Novel potent human ether-a-go-go-related gene (hERG) potassium channel enhancers and their *in vitro* antiarrhythmic activity. Mol. Pharmacol. 68, 876–884 (2005). 15976038 10.1124/mol.105.014035

[b31] Wang T. Z. *et al.* Muscarinic Receptor Activation Increases hERG Channel Expression through Phosphorylation of Ubiquitin Ligase Nedd4-2. Mol. Pharmacol. 85, 877–886 (2014). 24688054 10.1124/mol.113.091553

[b32] Potet F. *et al.* Identification and Characterization of a Compound That Protects Cardiac Tissue from Human Ether-a-go-go-related Gene (hERG)-related Drug-induced Arrhythmias. J. Biol. Chem. 287(2012). 10.1074/jbc.M112.380162 PMC3501040 23033485

[b33] Massey A. J. *et al.* A novel, small molecule inhibitor of Hsc70/Hsp70 potentiates Hsp90 inhibitor induced apoptosis in HCT116 colon carcinoma cells. Cancer Chemother. Pharmacol. 66, 535–545 (2010). 20012863 10.1007/s00280-009-1194-3

[b34] Smith J. L. *et al.* Trafficking-deficient hERG K(+) channels linked to long QT syndrome are regulated by a microtubule-dependent quality control compartment in the ER. Am. J. Physiol. Cell Physiol. 301, C75–85 (2011). 21490315 10.1152/ajpcell.00494.2010 PMC3129823

[b35] Zhang H. *et al.* Modulation of hERG potassium channel gating normalizes action potential duration prolonged by dysfunctional KCNQ1 potassium channel. Proc. Natl. Acad. Sci. USA 109, 11866–11871 (2012). 22745159 10.1073/pnas.1205266109 PMC3406814

[b36] Wu Z. Y., Chen K., Haendler B., McDonald T. V. & Bian J. S. Stimulation of N-terminal truncated isoform of androgen receptor stabilizes human ether-a-go-go-related gene-encoded potassium channel protein via activation of extracellular signal regulated kinase 1/2. Endocrinology 149, 5061–5069 (2008). 18599551 10.1210/en.2007-1802 PMC5398425

[b37] Kagan A., Yu Z. H., Fishman G. I. & McDonald T. V. The dominant negative LQT2 mutation A561V reduces wild-type HERG expression. J. Biol. Chem. 275, 11241–11248 (2000). 10753933 10.1074/jbc.275.15.11241

[b38] Foghsgaard L. *et al.* Cathepsin B acts as a dominant execution protease in tumor cell apoptosis induced by tumor necrosis factor. J. Cell Biol. 153, 999–1009 (2001). 11381085 10.1083/jcb.153.5.999 PMC2174340

[b39] Wang K. K. W. *et al.* An alpha-mercaptoacrylic acid derivative is a selective nonpeptide cell-permeable calpain inhibitor and is neuroprotective. Proc. Natl. Acad. Sci. USA 93, 6687–6692 (1996). 8692879 10.1073/pnas.93.13.6687 PMC39087

[b40] Withana N. P. *et al.* Cathepsin B inhibition limits bone metastasis in breast cancer. Cancer Res. 72, 1199–1209 (2012). 22266111 10.1158/0008-5472.CAN-11-2759 PMC3538126

[b41] Myers M. C., Shah P. P., Diamond S. L., Huryn D. M. & Smith A. B. 3rd. Identification and synthesis of a unique thiocarbazate cathepsin L inhibitor. Bioorg. Med. Chem. Lett. 18, 210–214 (2008). 18060772 10.1016/j.bmcl.2007.10.107 PMC2423727

[b42] Richardson P. G. *et al.* A phase 2 study of bortezomib in relapsed, refractory myeloma. N. Engl. J. Med. 348, 2609–2617 (2003). 12826635 10.1056/NEJMoa030288

[b43] Chapman H. *et al.* Downregulation of the HERG (KCNH2) K(+) channel by ceramide: evidence for ubiquitin-mediated lysosomal degradation. J. Cell Sci. 118, 5325–5334 (2005). 16263765 10.1242/jcs.02635

[b44] Phartiyal P., Sale H., Jones E. M. & Robertson G. A. Endoplasmic reticulum retention and rescue by heteromeric assembly regulate human ERG 1a/1b surface channel composition. J. Biol. Chem. 283, 3702–3707 (2008). 18048364 10.1074/jbc.M708999200

[b45] Rubio M. D., Wood K., Haroutunian V. & Meador-Woodruff J. H. Dysfunction of the ubiquitin proteasome and ubiquitin-like systems in schizophrenia. Neuropsychopharmacology 38, 1910–1920 (2013). 23571678 10.1038/npp.2013.84 PMC3746696

[b46] Bousman C. A. *et al.* Preliminary Evidence of Ubiquitin Proteasome System Dysregulation in Schizophrenia and Bipolar Disorder: Convergent Pathway Analysis Findings from Two Independent Samples. American Journal of Medical Genetics Part B-Neuropsychiatric Genetics 153B, 494–502 (2010). 10.1002/ajmg.b.31006 PMC4165610 19582768

