# Supplementary material for: Schizophrenia-Associated hERG channel Kv11.1-3.1 Exhibits a Unique Trafficking Deficit that is Rescued Through Proteasome Inhibition for High Throughput Screening
Source: Sci Rep. 2016 Feb 16;6:19976. doi: 10.1038/srep19976 (PMC4754628; doi:10.1038/srep19976)
Supplement: Supplementary Information [file srep19976-s1.pdf]

Supplementary Information:

Schizophrenia-Associated hERG channel Kv11.1-3.1 Exhibits a Unique Trafficking Deficit that is Rescued Through Proteasome Inhibition for High Throughput Screening

Nicholas E. Calcaterra[1], Daniel J. Hoeppner[2], Huijun Wei[2], Andrew E. Jaffe[2,3], Brady J. Maher[2,4,5*], and James C. Barrow[1,2*]

From the Departments of [1]Pharmacology and Molecular Sciences, [4]Psychiatry and Behavioral Sciences, and [5]Neuroscience, Johns Hopkins University School of Medicine, Baltimore MD 21205

[2]Lieber Institute for Brain Development, Baltimore, MD 21205

[3]Johns Hopkins University Bloomberg School of Public Health, Baltimore, MD 21205

Supplementary Table:

Literature compounds screened for Kv11.1-3.1 trafficking rescue

Compound	Description	Trafficking Rescue?	Selected References
**ALLN**	Calpain/Cathepsin/Proteasome inhibitor	Yes	22, 37
**Carbamoylcholine Chloride**	Cholinergic agonist	No	31
**Cisapride**	5HT-4 agonist/Cholinergic agonist/hERG inhibitor	No	29
**E4031**	hERG inhibitor	No	23, 29
**Mg132**	Proteasome inhibitor	No	36, 43
**ML-T531**	hERG activator	No	35
**Nocodazole**	Microtubule polymerization inhibitor	No	34
**PD-118057**	hERG activator	No	30
**VER-155008**	Hsp70 inhibitor	No	33
**VU-0405601**	hERG activator	No	32
